# APOS—antibiotic prophylaxis for preventing infectious complications in orthognathic surgery: study protocol for a phase III, multicentre, randomised, controlled, double blinded, clinical trial with two parallel study arms

**DOI:** 10.1186/s13063-021-05710-x

**Published:** 2021-11-02

**Authors:** Oliver Ristow, Christof Hofele, Philipp Münch, Sylvia Danner, Anja Dietzel, Johannes Krisam, Christina Klose, Maximilian Pilz, Jürgen Hoffmann, Christian Freudlsperger

**Affiliations:** 1 Department of Oral and Maxillofacial Surgery, University of Heidelberg, Im Neuenheimer Feld 400, D-69120 Heidelberg, Germany; 2 Coordination Centre for Clinical Trials (KKS), University of Heidelberg, 69120 Heidelberg, Germany; 3 Institute of Medical Biometry and Informatics, Department of Biometry, University of Heidelberg, Im Neuenheimer Feld 130.3, D-69120 Heidelberg, Germany

**Keywords:** Antibiotic prophylaxis, Surgical site infection, Randomised controlled trial, Orthognathic surgery

## Abstract

**Background:**

It is a constant debate among surgeons whether the use of prolonged postoperative antibiotics may reduce surgical site infection rates. As specific treatment guidelines are still lacking, many surgeons continue to use broad-spectrum antibiotics, causing not only increased costs but also contributing to the potential for antibiotic resistance. Hence, there is an urgent need for an appropriately designed prospective clinical trial, to investigate whether a prophylactic use of antibiotics after surgery actually decreases surgical site infections to a clinically relevant degree.

**Methods:**

This study presents a multicentre, randomised, controlled, double-blinded, clinical trial with two parallel study arms to demonstrate that no postoperative antibiotic prophylaxis (AP) is not inferior to antibiotic prophylaxis with respect to surgical site infections in patients having undergone orthognathic surgery. The primary efficacy endpoint is defined as the occurrence of postoperative surgical site infections within 30 days of surgery. Secondary endpoints are further efficacy and subject-oriented parameters within 90 days after surgery. The entire trial is planned for 54 months, with an enrolment of 1420 patients over 39 months by 14 national participating centres.

**Discussion:**

As a highly standardised procedure on an exceeding, healthy and young homogenous study population and identical processes all over the world, elective orthognathic surgery as clean-contaminated procedure provides comparable intervention groups with balanced baseline characteristics, comparable surgical duration, even when performed within multiple centres. Therefore, evaluating antibiotic prophylaxis after orthognathic surgery will be of high scientific value representable for other surgical procedures.

**Trial registration:**

DRKS—German Clinical Trials Register—DRKS00022838; EudraCT No. 2020-001397-30. Registered on 29 March 2021

## Administrative information

Note

The numbers in curly brackets in this protocol refer to SPIRIT checklist item numbers. The order of the items has been modified to group similar items (see http://www.equator-network.org/reporting-guidelines/spirit-2727-statement-defining-standard-protocol-items-for-clinical-trials/).

**Table:** 

Title {1}	APOS - Antibiotic Prophylaxis for preventing infectious complications in Orthognathic Surgery: Phase III, multicentre, randomised, controlled, double blinded, clinical trial with two parallel study arms
Trial registration {2a and 2b}.	DRKS - German Clinical Trials Register - DRKS00022838, registered 29. March 2021, https://www.drks.de EudraCT No. 2020-001397-30
Protocol version {3}	25-02-2021/Version 3
Funding {4}	Non-commercial study funded by DFG (Deutsche Forschungsgemeinschaft) GZ: RI2813/3-1
Author details {5a}	Oliver Ristow ^1^, Christof Hofele ^1^, Philipp Münch ^1^, Sylvia Danner ^2^, Anja Dietzel ^2^, Johannes Krisam ^3^, Maximillian Pilz ^3^, Kristina Klose ^3^, Jürgen Hoffmann ^1^, Christian Freudlsperger ^1^ 1 Department of Cranio-, Oral- and Maxillofacial Surgery, University Hospital Heidelberg, 69120 Heidelberg, Germany 2 Coordination Centre for Clinical Trials (KKS), University Hospital Heidelberg, 69120 Heidelberg, Germany 3 Institute of Medical Biometry and Informatics Department of Medical Biometry (IMBI), University Hospital Heidelberg, 69120 Heidelberg, Germany
Name and contact information for the trial sponsor {5b}	Ruprecht-Karls-University Heidelberg, Medical Faculty represented by Universitätsklinikum Heidelberg and Commercial Managing Director Katrin Erk, Im Neuenheimer Feld 672, 69120 Heidelberg Phone: 06221 / 56-7000, Fax: 06221 / 56-5925 E-Mail: Kaufmaennische-Direktion@med.uni-heidelberg.de
Role of sponsor {5c}	This is an investigator initiated clinical trial funded by the German Research Fundation (Deutsche Forschungsgemeinschaft, DFG); grant no.C RI2813/3-1. The funding body will not play any role in the trial.

## Introduction

### Background and rationale {6a}

Between 2000 and 2010, total global antibiotic consumption grew by more than 30% leading to increased resistance among common pathogens causing community and hospital-associated infections [1]. In hospitals, the suboptimal use of broad-spectrum and postsurgical antibiotics remains prevalent. While presurgical antibiotic prophylaxis (AP) is the evidence-based standard for preventing surgical site infections (SSI), no evidence supports the use of postoperative prophylactic antibiotics [2]. Note that although postoperative antibiotic application lasting longer than 24 h is considered as therapeutically approach [3], this change in terminology has not been respected in world literature over the last decades [4]. Currently, the amount of antibiotics given post-surgery is seven times higher as compared to pre-surgically administered antibiotics. This increases costs and contributes to the potential for antibiotic resistance [5]. However, many surgeons continue to use postoperative antibiotics while definitive treatment guidelines are still lacking.

Dentofacial deformities affect approximately 20% of the world’s population [6]. In 2012, about 10,000 patients have been hospitalised for orthognathic surgery (OS) alone in German university hospitals [7] not counting the approximately 10,000 OS which have been performed stationary on an outpatient basis. Direct communication of surgically mobilised osseous segments with the oral cavity, nasal cavity, or maxillary sinuses provides the basic rationale for the use of some type of antibiotic prophylaxis for these elective procedures (usually performed for 7 to 10 days), causing up to 160,000 postoperative antibiotic days per year [7]. However, there is a lack of evidence for the often-advocated antibiotic use after these elective procedures. Although a plethora of studies have been performed, there is no current consensus on the protocol for antibiotic prophylaxis after OS thus indicating the need for solid and reliable RCT-based evidence on this issue [4]. Clinically, postoperative infection may be associated with patient’s discomfort, prolonged hospital-stay and increases postoperative morbidity, which in turn leads to increased costs of medical care. Indeed, there is a sustained need to find out whether a prophylactic use of antibiotics after OS actually decreases SSI to a clinically relevant degree, especially in the context of increasing rates of resistance.

The declining efficacy of existing antibiotics potentially jeopardises outcomes in patients undergoing surgical procedures. There is an urgent need for new strategies and recommendations to reduce unspecified prophylactic antibiotics after surgical interventions throughout all fields of surgery, thus limiting the slowly upcoming catastrophe of increasing antibiotic resistances [8]. Thus, there is a serious lack of studies not solely evaluating SSI but also further outcomes that are important for clinical decision-making, such as systemic infection, duration of hospital stay as well as quality of life. Therefore, evaluating OS might be of high scientific value representable for other surgical procedures. As a highly standardised procedure with identical processes all over the world, elective OS provides comparable intervention groups with balanced baseline characteristics, comparable surgical duration and experienced and well-trained specialists, even when performed within multiple centres.

## Objectives {7}

### Primary objective

To demonstrate that no postoperative antibiotic prophylaxis is not inferior to antibiotic prophylaxis with respect to SSI in subjects having undergone OS.

The primary hypothesis is that the SSI rate in subjects undergoing OS without antibiotic prophylaxis (no AP) is not clinically relevant higher than in subjects with antibiotic prophylaxis (AP).

### Secondary objectives

To evaluate further efficacy and subject-oriented parameters of no AP in comparison to AP.

## Trial design {8}

Interventional, multicentre, randomised, controlled, double blinded trial with two parallel study arms. Subjects are allocated in a concealed fashion with a 1:1 ratio and a non-inferiority analysis will be performed for the primary endpoint.

## Methods: participants, interventions, and outcomes

### Study setting {9}

The clinical trial will be conducted on a national multicentre basis. Recruitment and treatment will take place at 14 experienced centres. Detail lists of study sites can be obtained at the registration form German Clinical Trials Register - DRKS00022838.

### Eligibility criteria {10}

As there will be no preferences on the selection of gender to be included, it is anticipated that the clinical trial results will give a representative gender distribution, which should reflect the natural gender distribution in the underlying disease.

#### Inclusion criteria

Subjects meeting all of the following criteria will be considered for admission to the clinical trial:

- Subject scheduled for elective, primary OS (bimaxillary or mandibular only approach)
- Age at study enrolment ≥ 18 years and < 65 years of age
- Ability of subject to understand character and individual consequences of the clinical trial
- Subject with basic literacy skills and ability to complete standardised health-related questionnaires
- Written informed consent (must be available before enrolment in the study)
- For women with childbearing potential and men capable of reproduction: agreement to remain abstinent (refrain from heterosexual intercourse) or use acceptable contraceptive methods in accordance with Clinical Trial Facilitation Group (CTFG) recommendation during treatment period with the investigational medical product (IMP) and for at least 1 day after the last dose of IMP. Women using hormonal contraception only agree to use an additional non-hormonal barrier method during and 1 day after the last dose of IMP. A woman is considered to be of childbearing potential, if she is postmenarcheal, has not reached a postmenopausal state (< 12 continuous months of amenorrhea with no identified cause other than menopause), and has not undergone surgical sterilisation (removal of ovaries and/or uterus)

#### Exclusion criteria

Subjects presenting with any of the following criteria will not be included in the clinical trial:

- Known hypersensitivity against Ampicillin/Sulbactam, other beta-lactam antibiotics and/or penicillin (and penicillin derivatives)
- Known hypersensitivity against per protocol proposed drugs for post-operative analgetic therapy (first- and second-line therapy, rescue medication: ibuprofen or diclofenac; metamizol; tilidin/naloxone or piritramid)
- Any condition in which elective surgery is not applicable
- Syndromal malformations
- Known renal insufficiency
- Known diabetes mellitus
- Current Morbus Pfeiffer disease
- Suffering from lymphatic leucemia
- Pregnancy or lactation
- Inability to comply with study and/or follow-up procedures
- Participation in another interventional trial

Exclusion criteria are defined considering risk of OS, as well as pharmacological effects and side effects to antibiotics according to the summary of product characteristics [9]. Subjects with a history of renal insufficiency or diabetes should be excluded to prohibit antibiotics or pain-drugs side effects. However, usually subjects scheduled for OS represent a healthy, young study population; therefore, this is expected to be very uncommon. Subjects with Morbus Pfeiffer disease or subjects suffering from lymphatic leucemia are excluded from the study based on the contraindications for ampicillin/sulbactam. No subject will be allowed to enrol in this clinical trial more than once.

### Who will take informed consent? {26a}

Informed consent will be obtained from every subject before the start of screening. One of the assigned study physicians of each participating centre will discuss the trial background, aims, potential risks and benefits with the subject. This includes collection and storage of personal and medical data from the subject for study purposes. The study physician will assure that all questions raised by the subject will be sufficiently answered. The subject can withdraw their consent at any time without reason. The informed consent sheet needs to be dated and signed by the subject and the study physician. If the subject is unable to write, oral presentation and explanation of the content of the informed consent form and of the data protection information must take place in the presence of an impartial witness. The witness and the physician conducting the informed consent discussions must also sign and personally date the consent document. The witness must not be in any way dependent on the sponsor of the clinical trial, the clinical trial site or any member of the investigating team (e.g. an employee at the clinical trial site). After eligibility criteria have been checked, the subject will be randomised into the trial.

### Additional consent provisions for collection and use of participant data and biological specimens {26b}

Additional collection of participant data and biological specimen are not planed.

## Interventions

### Explanation for the choice of comparators {6b}

As recommended for clean-contaminated surgeries, perioperative antibiotic prophylaxis is performed for all patients in the experimental as well as the control group following the standard protocol [3]. However, for the experimental and control intervention, different trials show no consensus regarding the optimal therapeutic strategy [4], and both interventions (no AP/AP after OS) are commonly used after OS worldwide.

The use of Ampicillin/Sulbactam is the standardised, broad-spectrum antibiotic for oral as well as head and neck associated infections [10]. The dosage of Ampicillin 1 g plus Sulbactam 0.5 g is recommended in the Summary of Product Characteristics (SmPC) [9] as well as the recommending societies [10–12]. Perioperative antibiotic prophylaxis is performed for all patients in a standard manner as specified by the corresponding guidelines (Ampicillin/Sulbactam 2/1 g iv.) during anaesthetic induction (30 min before incision) in order to achieve highest serum and tissue concentration of the substance at the time point of incision [3, 11, 12]. A repetition of the single dose application gets necessary, when operation time exceeds twice half-life period of the substance (half-life period Ampicillin/Sulbactam = 60 min). Therefore, refreshing is required after 2 h of surgical intervention.

Comparator intervention will receive Ampicillin/Sulbactam 1/0.5 g every 6–8 h iv. (according to manufacturer’s instructions) with 4 days of duration (respectively experimental, placebo application (NaCl 0.9% solution) will be performed for 4 days).

The postoperative use of Ampicillin/Sulbactam 1/0.5 g 1-1-1 iv. is the standardised antibiotic and application form for oral as well as head and neck associated infections [3, 10]. Furthermore, according to clinical practice, high dose Ampicillin/Sulbactam 2/1 g 1-1-1 iv. is supposed to be used in patients with severe infections under assumedly high bacterial load and in patients in critical clinical situation (e.g. sepsis patients with altered excretion parameters), whereas for non-severe infections clinicians more often choose lower dose [9, 10].

The APOS clinical trial population corresponds rather to the last group because they do not suffer from an evident infection. Furthermore, the three-times-a-day application of beta-lactam antibiotics, as planned for the APOS trial, is to prefer over a twice-a-day dosing. While the 12-h dosing scheme is mentioned in the SmPC for not-severe infections [9], the beta-lactam time-dependent pharmacokinetic (PK)/pharmacodynamics (PD) profile favours dosing every 8 h. Taking into account the PK profile of Ampicillin/Sulbactam, the minimum inhibitory concentrations (MIC) for bacteria, usually part of the oral flora, as gram-negative Anaerobes (MIC 4 mg/ml) or Streptococci (MIC 0.25 mg/ml) are expected to be exceeded in the plasma for at least 2 h and 6 h, respectively after an Ampicillin/Sulbactam dose of 1/0.5 g [9]. There are some data on oral tissue concentration of Ampicillin which indicate that Ampicillin/Sulbactam 1/0.5 g should be a sufficient dose for postoperative prophylaxis especially in the APOS trial population, where the perioperative Ampicillin/Sulbactam 2/1 g dose serves as loading dose [13, 14].

As mentioned before, until now, there is no evidence, and therefore no consensus on the most effective antibiotic regimen, concerning the length of postoperative application. In order to create strong evidence with a clear difference between the control and the experimental intervention, a long-term application was selected as long as necessary but as short as possible. After 4 days of antibiotic application a sufficient, broad activity spectrum is expected [9, 10]. Furthermore, after 4 days, sufficient closure of the surgical incision can be expected prohibiting the oral bacterial flora from freely entering the surgical site [15]. Application of antibiotics will be performed intravenously. After OS, oral intake is often complicated by intermaxillary fixation and postoperative morbidity. Furthermore, iv. application is the best form to control compliance and bioavailability and therefore to avert an attrition bias. All trial medication are prepared and blinded by the institutional pharmacies and delivered to the trial sites. After randomisation, trial medication has to be reconstituted by the local pharmacy according to manufacturer’s instructions. Physiologic salt solution will be used as placebo. Trial medication and placebo (both ready to use) will be blinded and labelled by the local pharmacy and then sent to the applicable trial site.

### Intervention description {11a}

Experimental intervention: no AP; placebo application (placebo NaCl solution 0.9% 1-1-1 iv.) after OS with 4 days of duration.

Control intervention: AP after OS (Ampicillin/Sulbactam 1/0.5 g 1-1-1 iv.; Proprietary Name: Unacid® or generic product ATC code: J01CR21) with 4 days of duration.

For clinical trial medication (verum/placebo) commercial products will be used in this study. In case the verum application Ampicillin/Sulbactam is not available in the dose of 1/0.5 g (e.g. due to supply shortage), Ampicillin/Sulbactam 2/1 g may be used alternatively for preparation of the infusion. If possible, the commercial product will be used in line with the local site’s standard.

Careful records will be kept of the commercial product purchased and administered as clinical trial medication to the subjects. If deficiencies of the clinical trial medication are noticed by the pharmacist or study personnel, the coordinating investigator and the monitor and/or project manager must be informed immediately.

The administration of the infusion will be carried out by study staff as delegated by the investigator and according to the applicable regulations and local rules of the institution.

The clinical trial medication is given only to subjects who have consented to clinical trial participation. Duration of treatment is as follows: treatment will start on the day of surgery and will end on postoperative day (POD) 4. A total of 12 doses will be administered to the subjects during treatment phase over a maximum time period of 96 h. The first dose of study medication will be administered postoperatively the latest in the evening of surgery day. On the following three days (POD1, POD2, POD3), subjects will receive three daily doses with clinical trial medication (dosing interval 6–8 h or according to manufacturer’s instructions). On POD4, subjects will receive the remaining infusions to have administered a total of 12 doses.

Subjects will receive an infusion of Ampicillin/Sulbactam prepared from Ampicillin/Sulbactam 1/0.5 g powder or the placebo application depending on the result of randomisation. Physiological salt solution (Sodium Chloride 0.9 %) will be used as placebo. The infusion time will be 15–30 min for both applications. Further instructions will be provided in a pharmacy manual.

### Criteria for discontinuing or modifying allocated interventions {11b}

A subject will be withdrawn from treatment, if any of the below reasons apply (one primary reason must be determined):

- Intolerable adverse events, i.e. a medical event which puts the subject at risk of relevant or persistent health deterioration if the trial treatment will be continued, e.g.:
  - Severe acute hypersensitivity symptoms (facial oedema, swelling of the tongue, internal swelling of the larynx with narrowing of the airways, heart palpitations, shortness of breath, serum sickness, allergic inflammation of the blood vessels (allergic vasculitis), eosinophilia, haemolytic anaemia, nephritis, drop in blood pressure, anaphylactic/anaphylactoid reaction, anaphylactic/anaphylactoid shock)
  - Severe skin reactions such as erythema multiforme, Stevens-Johnson syndrome or toxic epidermal necrolysis, acute generalised inflammatory rash with blistering (acute generalised exanthematic pustulose), skin inflammation with scaling and flaking of the skin (exfoliative dermatitis), inflammatory reaction of the skin (dermatitis)
- Treatment with drugs with known drug-drug interactions according to the applicable SmPC for ampicillin/sulbactam:
  - Antibiotics or bacteriostatic chemotherapeutics (e.g. tetracyclines, erythromycin, sulfonamides, chloramphenicol)
  - Allopurinol, methotrexate, probenecid
  - Anticoagulants: If treatment with anticoagulants becomes medically necessary, the decision whether the subject should be withdrawn from further treatment with IMP depends on the decision of the investigator
- Lack of subject’s cooperation, e.g.
  - Subject’s request to withdraw from treatment
  - Lack of compliance
  - Intended pregnancy, lactation
- Pregnancy
- Any medical condition the investigator or the sponsor deems as a risk to the subject and subject’s safety when continuing with study treatment
- Decision on the part of the Investigator or Sponsor that withdrawal from the treatment is in the patient´s best interest
- Other reasons (noting reason), e.g.
  - Other diagnosis than clinical trial disease
  - Intercurrent illness (inclusive the development of conditions listed in exclusion criteria) which interferes with trial objectives or puts the subject at risk of relevant or persistent health deterioration if immediate measures interfering with the trial course or its objectives will not be introduced (e.g. post-operative treatment with corticosteroids)
  - Did not meet major in-/exclusion criteria (coming to light after randomisation)

### Strategies to improve adherence to interventions {11c}

As the trial medication will be administered as infusion to the subjects during hospitalisation, it is not necessary to check the subject’s compliance. The preparation and administration of the infusions will be documented on appropriate forms by the pharmacy and site staff and infusions will be recorded in the eCRF.

### Relevant concomitant care permitted or prohibited during the trial {11d}

During surgery, all subjects will receive a prophylactive administration of 4 mg dexamethasone in order to prevent postoperative nausea and vomiting (PONV) [16]. No further applications with corticosteroids are allowed during the study.

According to each centre’s local standards all subjects will receive ice pack application or Hilotherm® after surgery during hospitalisation.

Postoperative analgesic drug therapy will be performed as a standard regimen for all subjects:

- First line drug: Ibuprofen 600 mg parenteral (three times daily) or Diclofenac disp. 50 mg (three times daily) till discharge and if further needed till POD30
- Second line drug: Metamizol-Natriummonohydrat up to 1 g (three times daily) till POD4, if further needed till POD30
- Rescue drug: tilidin/naloxone 50/4 mg up to three times daily or piritramid 3.75–7.5 mg in 100 ml NaCl administered as short infusion up to three times daily, if further needed till POD4

Dose and duration of postoperative analgesic drug therapy will be documented in the patient medical file and electronic case report file (eCRF) until POD30.

In addition to usual oral hygiene procedures, all subjects will receive chlorhexidine 0.12 % for rinsing three times daily for 10 days after surgery till POD10.

The treatment of accompanying illnesses not subject to the exclusion criteria is allowed, if this is not expected to have any effect on the outcome measures used in this clinical trial and to interfere with the clinical trial medication.

In particular, the following drug groups are not permitted as concomitant medication:

- Corticosteroids

With exception of the standard prophylactic administration of 4 mg dexamethasone during surgery, all further applications of corticosteroids will be explicitly excluded from the study protocol, since they are not a standardised and necessary regime after OS.

- The following drugs with known drug-drug interactions according to the applicable SmPC are not permitted during treatment with the IMP:
  - Antibiotics or bacteriostatic chemotherapeutics (e.g. tetracyclines, erythromycin, sulfonamides or chloramphenicol)
  - Allopurinol, methotrexate, probenecid

Anticoagulants: If medically required after surgery, anticoagulants are permitted depending on the decision of the investigator.

If the subject develops any symptoms or diseases postoperatively during or after hospitalisation and requires concomitant medication not permitted within this study, this will not lead to withdrawal from the study, and the subject will be further assessed within the study. The treatment of accompanying symptoms or diseases is allowed at any time, if medically imperative.

If concomitant drugs are administered, these must be recorded in the subject file and in the eCRF, stating

- International Nonproprietary Name (INN)
- Indication for use
- Start/end date.

Existing, permitted concomitant treatments are not to be changed during the course of this clinical trial, unless medically required.

### Provisions for post-trial care {30}

Planned treatment after clinical trial end: The investigator will continue to observe all subjects (also withdrawals) because of intolerable AEs/ SAEs until the findings have been resolved or became stable. For injuries caused to participating persons and arising out of this research performed in accordance with the protocol and applicable law and professional standards, an insurance is in place.

## Outcomes {12}

### Primary efficacy endpoint

Occurrence of postoperative SSI within 30 days after surgery (POD30). SSI is the most common and relevant complication after OS and may be associated with subject’s discomfort, prolonged hospital stay, increased postoperative morbidity, and higher costs of medical care. Thus, SSI has been evaluated in different previous trials [4]. Definition and determination of SSI are based on the standardised used criteria of the American “Center for Disease Control and Prevention” (CDC), which correspond to the recommendations of the “European Center for Disease Prevention and Control (ECDC) as well as the recently introduced German KISS-definitions (Krankenhaus-Infektions-Surveillance-System) of the “National reference centre for surveillance of nosocomial infections - Robert Koch Institut” [17–19]. Therefore, those criteria are the worldwide absolute, undisputed standard in the assessment of postoperative SSI. All of those criteria were introduced to guarantee international comparability of surveillance data and have been used in a plethora of international recognised studies [4, 15, 20]. Just recently, the new German KISS-definitions (which are in form and content based on the CDC criteria) for the evaluation and prevention of SSI have been published [17, 18].

### Secondary endpoints

1. Deep incisional and/or organ or space SSI as defined by CDC/KISS [17, 21] 30 (POD30) to 90 days after surgery (POD90). As an implant is used, a deep incisional or organ and space SSI may occur within 90 days after surgery according to CDC/KISS criteria [17, 21].
2. Systemic infections, defined as a systemic inflammatory response syndrome associated with a postoperative SSI consecutive to OS. Systemic infections might be associated with postoperative SSI. However, since OS is a scheduled procedure, and surgeons do not perform it in patients at high risk of infection, systemic infection is very unlikely to occur.
3. Length of hospital stay (LOS), defined as the number of days from OS to the day of discharge. LOS is an important subject-oriented outcome and can be directly or indirectly influenced by SSI.
4. Health-related quality of life (HRQoL), measured by SF-36 and OHIP-G-14 [22–24]. HRQoL is an important patient-oriented outcome and can be directly or indirectly influenced by SSI. Furthermore, there might be connections between subjects’ expectations regarding the postoperative AP and their QoL independent from an SSI. Antibiotics are often prescribed due to subjects’ expectations in order to enhance their QoL.
5. Non-severe medication related adverse events (AE), defined as mild diarrhoea or skin rash due to antibiotic administrations. Ampicillin/Sulbactam antibiotic are well established, tested and tolerated antibiotics. However, diarrhoea and skin rashes are reported. Adverse effects associated with antibiotic intake are of great interest when evaluating the elective/prophylactic use of antibiotics.

### Participant timeline {13}

Table 1 shows the clinical trial schedule. The clinical trial period for an individual subject consists of a 4-day treatment period and a follow-up phase until POD90.

**Table 1:** Clinical trial schedule

Visit no.		V1	V2			V3		V4	V5	V6	V7	V8
Phase		Screening/baseline		Treatment					Follow up			
Relative time point	Day	D-28 to D-1 Pre-surgery visit/hospital admission^a^	D0		POD1	POD2	POD3	POD4	POD 10 ± 3 days	POD 30 ± 3 days	POD 60 ± 3 days	POD 90 ± 3 days
			Pre-OS	Pos-OS								
Procedure												
Inclusion/ exclusion criteria	C	●										
Written informed consent	C	●										
Medical history, demography	R	^e^●										
Randomisation	C	^b^●										
Surgery			●									
^d^IMP administration	C			●	●	●	●	●				
Examination of mouth cavity	R	^e^●										
Mouth hygiene	R	●				●		●	●	●	●	●
^f^SSI	R					●		●	●	●	●	●
^g^Pregnancy test	C	^c^●										
^h^Blood tests: CRP, WBC	R	^c^●						●		●		
HRQoL:SF-36, OHIP-G-14	C	●						●		●		●
^i^AE	R			●	●	●	●	●	●	●	●	●
^j^Concomitant medication	R	●		●	●	●	●	●	●	●	●	●
^k^Postoperative pain medication	R			●	●	●	●	●	●	●		
Smoking and/or alcohol consumption	R	●				●	●	●	●	●	●	●

*R* regular or *C* clinical trial specific examination; *POD* postoperative day; *OS* orthognathic surgery; *V* visit

^a^Routine pre-surgery information visit approx. 3–4 weeks before OS; routine hospital admission: D-1

^b^Randomisation: D-1 or earlier when subject eligibility confirmed and IC obtained

^c^Lab tests at hospital admission D-1

^d^IMP administration: Total of 12 doses will be administered during treatment phase (D0–POD4) over a period of 96 h

^e^Pathological findings detected prior to the 1st administration of IMP are to be documented as medical history, thereafter as adverse events

^f^SSI: post-op. evaluation of mouth cavity for signs and symptoms for SSI

^g^In females of child-bearing potential; performed in blood (HCG) or urine

^h^Blood tests: C-reactive protein (CRP) and WBC count. If SSI is diagnosed independent of trial visits, an additional blood sample is taken

^i^AE/SAE: adverse/serious adverse events. Pathological findings detected at/after the first administration of IMP are to be documented as AE/SAE

^j^Concomitant medication: prior concomitant medication (inclusive pre-operative medication, exclusive intraoperative medication)

^k^Postoperative pain medication as needed till POD30 to be documented

### Sample size {14}

The sample size calculation is based on the primary efficacy endpoint “Occurrence of SSI within thirty days after surgery (V4)”. A recent meta-analysis [1] reported a pooled risk ratio of 0.42 (95% confidence interval: [0.24; 0.74]) for the comparison of long-term (before or during surgery and longer than one day after surgery) versus short-term (before or during surgery and/or during the same day) antibiotic prophylaxis. Furthermore, pooled event rates of 7.1% for long-term and 16.8% for short-term antibiotic prophylaxis were observed, yielding a difference of 9.7%. Thus, for both the AP and no AP group, an infection rate of 7.1% is expected, as the assumption of equal rates is the standard approach for a power calculation in non-inferiority trials. If the rate in the no AP group is at most 4% higher than the rate in the AP group, this will be considered as clinically irrelevant since possible SSI might have an increase in clinically manageable morbidity, however, no life-threatening consequences for subjects.

Based on these assumptions, a total of *n* = 1346 subjects (673 per group) are required to demonstrate non-inferiority at a margin of *δ* = 4% for no AP as compared to AP applying the Farrington-Manning test at a one-sided significance level of *α* = 2.5% with a power of 1-β = 80% (calculations performed with ADDPLAN v6.1). The adjustment for the factor centre using a Mantel-Haenszel type test is expected to yield an additional increase in power. To account for a drop-out-rate of 5%, a total of *n* = 1420 subjects (710 per group) will be randomised.

The potential problem of missing values for the primary outcome due to loss to follow up or premature withdrawal from the study before the measurement of the primary endpoint will be partly resolved in the confirmatory analysis by application of a pre-defined imputation strategy (see the “Statistical methods” section).

### Recruitment {15}

The clinical trial will be conducted on a multicentre basis. Recruitment and treatment will take place at 14 centres. We calculated that 1420 subjects are being assigned for this trial within a recruitment period of 39 months resulting in approx. 570 subjects per year. In order to prove the feasibility of recruitment, we screened the DRG flat-rate catalogue of the German federal statistical office [7], the DRG index benchmarking reports of the university departments for oral and maxillofacial surgery in Germany as well as the results of the QB-analyser [25]. Justified on the department’s performance, invitation to participate was sent to the eligible centres. The coordinating centre at Heidelberg University conducts more than 200 OS per year, of which approximately 150 will are estimated to meet the inclusion criteria and agree to participate in the trial. With a major percentage of all OS performed on German Universities, the applicants will be able to contribute a large share of the necessary enrolment. Thirteen other centres with great expertise in OS, and thereby fulfilling the criterion for participation, have signed agreement to participate. According to each centre’s declaration of commitment, recruitment of 1420 subjects is expected to be feasible within 39 months. According to our experience, since all surgical interventions are elective on usually young and healthy subjects, there is a motivation to take part in trials aiming to reduce antibiotic intake.

## Assignment of interventions: allocation

### Sequence generation {16a}

To achieve comparable intervention groups, subjects will be allocated in a concealed fashion in a 1:1 ratio by means of randomisation which will be performed by delegated staff members of the local pharmacy using a centralised web-based tool (www.randomizer.at). Randomisation will be performed stratified by location (bimaxillary or mandibular only osteotomy) and centre. Block randomisation will be performed to achieve in total equal group sizes per centre and location.

### Concealment mechanism {16b}

Details of the randomisation scheme such as the block length are specified by the trial biometrician and only provided in an external document accessible to the Institute of Medical Biometry and Informatics (IMBI) only to minimise selection bias. The block length will not be disclosed to any other personnel involved in the trial, thus providing allocation concealment. The randomisation numbers will be allocated sequentially in the order in which the subjects are enrolled.

### Implementation {16c}

In each clinical trial site, all subjects who enter the screening period for the trial will receive a unique Subject ID before any trial-related procedure is performed. The Subject ID (Screening Number) will be assigned by the clinical trial site as a consecutive number. This Screening Number will be used to identify the subject throughout the clinical trial and must be used on all trial documentation related to the patient. Clinical sites must complete the appropriate pages of the electronic case report form (eCRF) for all subjects randomised, even if the subject is not treated in the study. Subjects who fulfil the enrolment criteria—which will be checked by the clinical site—will be randomised by the pharmacy or a delegated staff member not involved in the assessment of the subject. The allocation sequence is determined via the employed online randomisation tool based on the specifications provided by the trial biometrician. The Screening Number will be entered as Subject-ID into the randomisation tool which will allocate the Randomisation Number sequentially in the order in which the subjects have been enrolled. Both the Screening Number and the Randomisation Number will be recorded on the individually prepared infusion bag by the pharmacist or a delegated site member not involved in the assessment of the subject. Every site is obliged to strictly adhere to this randomisation procedure. The correct assignment will also be verified by the clinical monitor at each site.

## Assignment of interventions: blinding

### Who will be blinded {17a}

Double blinding of investigators and subjects will be performed in order to avoid bias due to differences in performance and perception. In addition to the clinical trial medication prepared by the local pharmacy after randomisation of each patient, a sealed envelope (emergency envelope) will be forwarded to the investigator via the local pharmacy, one for each randomisation number. The envelope contains information on the subject’s clinical trial medication and is to be opened only under circumstances in which it is medically imperative to know what the subject is receiving. The envelopes are not to be opened by the investigator at the end of the clinical trial. All envelopes will be collected by the responsible monitor at the end of the clinical trial.

### Procedure for unblinding if needed {17b}

Only delegated members of the local pharmacy (or other staff member not involved in the subjects’ treatment and assessment as delegated by the investigator, ensuring that study staff will at all times remain blinded regarding subject’s treatment) responsible for performing randomisation at the site will have access to the randomisation tool. If it is medically imperative to know what clinical trial medication the subject is receiving, the investigator or authorised person should open the emergency envelope. The investigator or the authorised person who breaks the blind must record the date and the reasons for doing so in the eCRF, in the subject’s medical record and on the emergency envelope. Whenever possible, the coordinating investigator (LKP) should be contacted before the blind is broken.

## Data collection and management

### Plans for assessment and collection of outcomes {18a}

All protocol-required information collected during the trial must be entered by the investigator, or designated representative, in the eCRF. The investigator, or designated representative, should complete the eCRF pages as soon as possible after information is collected, preferably on the same day that a trial subject is seen for an examination, treatment, or any other trial procedure. Any outstanding entries must be completed immediately after the final examination. An explanation should be given for all missing data. The completed eCRF must be reviewed and signed by the investigator named in the trial protocol or by a designated sub-investigator.

### Plans to promote participant retention and complete follow-up {18b}

The patients will receive extensive information about the study set-up and requirements during the recruitment. The importance of completion of the follow up will be stressed. However, the frequency of visits is consistent with the routine clinical care of this subject group. All measurement time points chosen are part of the standardised follow-up protocols and standard visits after OS interventions. The scheduled investigations are consistent with routine clinical care and partly correspond to study-related procedures. Since the intervention always is a combination of surgery and orthodontics, with a succeeding period of therapy by the orthodontics up to 2 years after surgery, compliance is very high in OS patients and loss of follow-up is expected to be low.

### Data management {19}

The IMBI is responsible for the data management within the trial. An eCRF will be used for data collection. To assure a safe and secure environment for data acquired, data transmission is encrypted with secure socket layer (SSL) technology. Only authorised users are able to enter or edit data, the access is restricted to data of the subjects in the respective centre. All changes to data are logged with a computerised timestamp in an audit trail. All data will be pseudonymised. To guarantee high data quality, data validation rules will be defined in a data validation plan. Completeness, validity and plausibility of data will be checked in time of data entry (edit-checks) and using validating programs, which will generate queries. If no further corrections are to be made in the database, eCRF data will be locked. Data will finally be downloaded and used for statistical analysis. All data management procedures will be conducted according to written defined standard operating procedures (SOPs) of the IMBI that guarantee an efficient conduct complying with good clinical practice (GCP). At the end of the study, the data will be transformed into different data formats for archiving and to ensure that it can be re-used.

### Confidentiality {27}

The data obtained in the course of the clinical trial will be treated pursuant to the EU General Data Protection Regulation (GDPR) (national regulatory requirements, e.g. (Bundesdatenschutzgesetz, BDSG).

During the clinical trial, subjects will be identified solely by means of their individual identification code (screening number, randomisation number). Clinical trial data stored on a computer will be stored in accordance with local data protection law and will be handled in strictest confidence. Distribution of these data to unauthorised persons has to be prevented strictly. The appropriate regulations of local data legislation will be fulfilled in its entirety.

The subject consents in writing to release the investigator from his/her professional discretion in so far as to allow inspection of original data for monitoring purposes by health authorities and authorised persons (inspectors, clinical monitors, auditors). Authorised persons (inspectors, clinical monitors, auditors) may inspect the subject-related data collected during the clinical trial ensuring compliance with the effective data protection law.

The investigator will maintain a subject identification list (screening numbers with the corresponding subject names) to enable records to be identified. Subjects who did not consent to circulate their pseudonymised data will not be included into the clinical trial.

This protocol, the eCRFs and other clinical trial-related documents and material must be handled with strict confidentiality and not be disclosed to third parties except with the express prior consent of Sponsor. In particular, it must be ensured that the clinical trial medication is kept out of reach of third parties. Staffs of the investigators involved in this clinical trial are also bound by this agreement.

### Plans for collection, laboratory evaluation and storage of biological specimens for genetic or molecular analysis in this trial/future use {33}

There will be no collection of biologic specimens for further analysis in the future in this trial.

## Statistical methods

### Statistical methods for primary and secondary outcomes {20a}

#### Analysis of primary endpoint

Non-inferiority of no AP vs. AP will be assessed using the test according to Mantel and Haenszel extended to the non-inferiority case [26, 27] which allows adjustment for the factor centre. The one-sided significance level is set to 2.5%. The hypotheses to be assessed in the primary efficacy analysis are as follows: *H*_0_ : *p*_*no AP*_ − *p*_*AP*_ ≥ *δ* and *H*_1_ : *p*_*no AP*_ − *p*_*AP*_ < *δ*, where *δ* = 4% represents the chosen non-inferiority margin and *p*_*no AP*_ / *p*_*AP*_ denote the SSI rate at Visit 4 (POD30) in the no AP and AP group, respectively.

The primary efficacy analysis will be based on the modified ITT set including all randomised subjects who postoperatively received at least one dose of placebo or AP, reflecting the recommendations given in guidelines (e.g. [28]). Furthermore, in [29], it was demonstrated that for the particular setting of antibiotic non-inferiority trials, the ITT and the PP set, excluding subjects with major protocol violations, yield almost identical results. Thus, modified ITT should be preferred over PP as the primary efficacy set here. Additionally, an evaluation of the primary outcome will be performed in the PP set, in accordance to [30] where the importance of the PP set in non-inferiority trials was emphasised. Before database closure, the assignment of each subject to the modified ITT and the PP population are defined in the statistical analysis plan.

Sensitivity analyses will be performed by applying alternative methods dealing with missing data such as, e.g. complete case analysis and replacement by ICA-r [31]. Besides the adjustment for centre which is included into the primary analysis, a generalised linear model with identity link and binomial error will additionally be fitted for a comparison of the SSI rates between the groups adjusting for location and centre. Furthermore, the primary endpoint will be evaluated by the Farrington-Manning test [32] for the unadjusted non-inferiority assessment of rate differences not considering any potential confounders.

#### Analysis of secondary endpoints

All secondary outcomes will be evaluated descriptively. Analyses of secondary endpoints will be conducted based on the m-ITT set, with additional sensitivity analyses based on the PP set.

The secondary endpoints “Deep incisional and/or organ or space SSI within 90 days after surgery” and the secondary endpoint “systemic infections” will be evaluated by means of a logistic mixed regression model using for the fixed factors treatment group and location, as well as the random factor centre. Odds ratios will be given with 95% confidence intervals together with descriptive *p* values.

The secondary endpoint LOS will be assessed using Kaplan-Meier estimates for each treatment group. A Cox regression frailty model with the fixed factors treatment group and location and the random factor centre will be fitted. A hazard ratio for the factor treatment group will be given with a 95% confidence interval together with a descriptive *p* value.

HRQoL at follow-up will be analysed using a linear mixed model for repeated measurements, taking into account the HRQoL baseline value, treatment group, and location as fixed factors, centre as random factor, and time as repeated factor using an unstructured covariance matrix. 95% confidence for the mean difference between treatment groups will be given with descriptive *p* values. No imputation of missing data will be done for secondary endpoints.

Furthermore, the treatment effect will be assessed descriptively within several subgroups of interest to identify potential prognostic and predictive factors.

The safety analysis includes calculation of frequencies and rates of adverse and serious adverse events together with 95% Wilson-type confidence intervals. Safety analysis will be based on the safety set comprising all subjects of the m-ITT set who postoperatively received at least one dose of placebo or postoperative AP and will allocate the subjects to the treatment they actually received, regardless of randomisation.

Statistical analysis is based on the International Conference on Harmonization (ICH) Guidelines “Structure and Content of Clinical Study Reports” and “Statistical Principles for Clinical Trials” [28]. All statistical procedures are done according to the current standard operating procedures (SOPs) of the Institute of Medical Biometry and Informatics, University of Heidelberg (IMBI).

### Interim analyses {21b}

Not applicable since no interim analyses are planned.

### Methods for additional analyses (e.g. subgroup analyses) {20b}

Descriptive analyses of primary and secondary endpoints will be done within several subgroups of interest to identify potential prognostic and predictive factors. The detailed methodology for all statistical analyses is described in the statistical analysis plan (SAP), which is finalised before database lock. Statistical analysis is performed using SAS v9.4 or higher.

### Methods in analysis to handle protocol non-adherence and any statistical methods to handle missing data {20c}

Missing data for the primary outcome variable due to loss to follow-up or early withdrawal before measurement of the primary endpoint will be replaced by using multiple imputation [33], which takes the covariates treatment group, location (bimaxillary or mandibular osteotomy), and centre into account by application of the fully conditional specification method [29]. This will be realised using the option “FCS” of the SAS “MI” procedure which is implemented in SAS 9.4.

Post-randomisation events will be handled as follows in accordance with the estimands framework. Subjects not undergoing surgery or not receiving at least one dose of postoperative AP or placebo will be excluded, which corresponds to a modified intention-to-treat principle and a principal stratum strategy, as not undergoing surgery or not receiving at least one dose of study treatment is independent of treatment allocation due to the blinded character of the trial. Patients who are lost to follow up or prematurely withdraw their consent before measurement of the primary endpoint will be considered as missing and multiple imputation will be used to impute the primary outcome of these patients, corresponding to a hypothetical strategy. Besides these events, other post-randomisation events will not be considered, thus reflecting a treatment policy approach, which means that the effect of randomised treatment is estimated irrespectively of other post-randomisation events not captured in the primary endpoint definition.

### Plans to give access to the full protocol, participant level-data and statistical code {31c}

The datasets used and/or analysed during the current study can be made available by the corresponding author upon reasonable request and in agreement with the research collaboration and data transfer guidelines of the University of Heidelberg.

## Oversight and monitoring

### Composition of the coordinating centre and trial steering committee {5d}

The steering committee (SC) is comprised of the coordinating investigator and his supporting co-investigators, clinical experts not directly involved in the clinical trial and the responsible biometrician. The SC is responsible for the scientific integrity of the clinical trial protocol, the quality of the clinical trial conduct as well as for the quality of the final clinical trial report. The SC will decide on the recommendations made by the DMC.

### Composition of the data monitoring committee, its role and reporting structure {21a}

A Data Safety Monitoring Board (DSMB) will be set up to monitor safety aspects of the trial and to support the sponsor’s decision-making regarding progress of the trial. The working methods of the committee are based on the guideline EMEA/CHMP/EWP/5872/03 Corr.

The tasks of the DSMB are to ensure the ethical conduct of the trial and to protect the rights and welfare of the involved patients. The DSMB consists of 3 experts representing the field of cranio-, oral and maxillo-facial surgery and statistics. Since the trial is carried out in a double-blind fashion, an external biometrician participates in the DSMB. The DSMB will be regularly informed of all safety aspects of the trial and will review the safety data. A meeting or telephone conference with the board will be scheduled approximately twice a year to review the trial’s progress, to ensure adherence to the protocol, to advise whether to continue, modify, or stop the trial. The DSMB will also assess whether or not the recruitment plan is on target. Based on relevant information from within the trial or from other sources, the DSMB will monitor possible (serious) adverse events and may make a recommendation to the sponsor or to the SC to stop the trial at any time.

### Adverse event reporting and harms {22}

AEs will be ascertained by the investigators using non-leading questions, noted as spontaneously reported by the subjects to the medical staff or observed during any measurements on all clinical trial days. The observational period begins with the first administration of the IMP (day 0, postoperatively) and ends with the visit 8, POD90. However, all AEs observed up to POD90 will be followed up in order to determine their outcome. AEs will be documented in the subject file and in the eCRF. All AEs untoward medical events occurring prior to the beginning of the observational period will be recorded in the eCRF as medical history. All subjects who present AEs, whether considered associated with the use of the clinical trial medication or not, will be monitored by the responsible investigator to determine their outcome. This applies also to subjects who were withdrawn from the clinical trial.

The subjects should report any AEs occurring during the outsubject part to the clinical trial centre by phone.

All SAEs and their relevance for the benefit-risk assessment of the clinical trial will be evaluated continuously during the clinical trial and for the final report. All SAEs will be documented in the “Serious Adverse Event” form and must be reported by the investigator to the delegated Safety Department immediately, but not later than 24 h after the SAE becomes known. All SAEs will be subject to a second assessment by Sponsor’s designated person, who will be independent from the reporting investigator.

Pregnancy itself is not regarded as an adverse event. If a subject becomes pregnant during the course of the clinical trial, the treatment must immediately be discontinued. The outcome of any conception occurring between the first administration and 3 months after the last administration of the IMP will be followed up and documented. If any pregnancy, suspected pregnancy, or positive pregnancy test occurs in the course of the clinical trial, it must immediately be reported to the delegated Safety Department.

### Frequency and plans for auditing trial conduct {23}

Regulatory authorities and/ or auditors authorised by the sponsor may request access to all source documents, eCRFs, and other clinical trial documentation. Direct access to these documents must be guaranteed by the investigator who must provide support at all times for these activities. The investigator will inform the sponsor immediately about a planned inspection.

### Plans for communicating important protocol amendments to relevant parties (e.g. trial participants, ethical committees) {25}

Before the start of the clinical trial, the clinical trial protocol, informed consent document, and any other appropriate documents will be submitted to the independent Ethics Committee (EC) as well as to the competent authority (BfArM).

A written favourable vote of the EC and an (implicit) approval by the competent authority are a prerequisite for initiation of this clinical trial. The statement of EC should contain the title of the clinical trial, the clinical trial code, the clinical trial site, and a list of reviewed documents. It must mention the date on which the decision was made and must be officially signed by a committee member. This documentation must also include a list of members of the EC present on the applicable EC meeting and a GCP compliance statement.

Coordination Centre for Clinical Trials (KKS) Heidelberg who is responsible for submitting the documents will keep a record of all communication with the EC and the regulatory authorities. Before the first subject is enrolled in the clinical trial, all ethical and legal requirements must be met. All planned substantial changes (see §10, [1] of German GCP-Ordinance) will be submitted to the EC and the competent authority (BfArM) in writing as protocol amendments. They have to be signed by the sponsor and biometrician and approved by the EC and the competent authority.

### Dissemination plans {31a}

All information concerning the clinical trial is confidential before publication. Publication(s) and/or presentation(s) of the clinical trial results is encouraged after appropriate time for review and written agreement by the sponsor. The sponsor has to be provided with a draft of the abstract and/or manuscript for review and editorial comments at least 30 days prior to submission and/or presentation. Neither the sponsor nor the Coordinating Investigator has the right to prevent publication, except for patent or copyright purposes. Clinical trial data published or disclosed to third parties must not contain data that allow the identification of a subject.

## Discussion

OS is the surgical correction of a deformity of the jaw. It is a general term that includes several elective surgical techniques to correct facial deformity, the associated malocclusion and functional disorders related to the stomatognathic system [34]. Compared to other surgical procedures, OS are elective, worldwide standardised and therefore reproducible techniques that are usually performed by the same surgical teams, always in a stationary setting. Because the upper digestive tract is penetrated during OS, interventions are classified as clean-contaminated [35] and consequently AP might be mandatory in order to prevent surgical site infection when implant insertions are being performed.

Indeed, the role of bacterial biofilms from the surface of implants (as used for stabilisation of maxillary or mandible osteotomy segments) in the development of local or regional infectious complications is well recognised and has been evaluated in many experimental studies [36]. Some study authors advocate that morbidity can be kept to a minimum with adherence to general surgical principles [8]. Additionally, it is hypothesised that prophylactic antibiotics are of questionable value regarding the prevention of infections, and that their deployment could lead to the development of super infections and stimulate further resistances to antibiotics [1].

Hence, it is discussed whether the use of prophylactic antibiotics may reduce the postoperative infection rates [2]. Therefore, many attempts have been made to determine the efficacy of AP after OS and highly heterogeneous results have led to a plethora of inconsistent recommendations within the literature [4]. Even systematic reviews incorporating meta-analyses focusing on this issue vary in their conclusions [37].

This shortcoming motivated the Cochrane collaboration to perform a systematic review in 2015 including 11 (out of 96 eligible) studies. An overall moderate quality of evidence was identified, thus uncovering the need to perform a RCT in which additional outcomes besides surgical site infections are assessed [4]. However, current prospective RCTs fail Cochrane’s appeal in respect to the study protocol, power estimation, bias and medication chosen [15]. In 2018, a newly performed meta-analysis highlights again this shortcoming and concludes that in the field of OS, most studies of antibiotic prophylaxis are poorly performed and reported [20]. Therefore, scientific uncertainty remains with respect to the necessity, preferred antibiotic compound and optimal range of the prophylaxis.

## Trial status

**Fig. 1:**
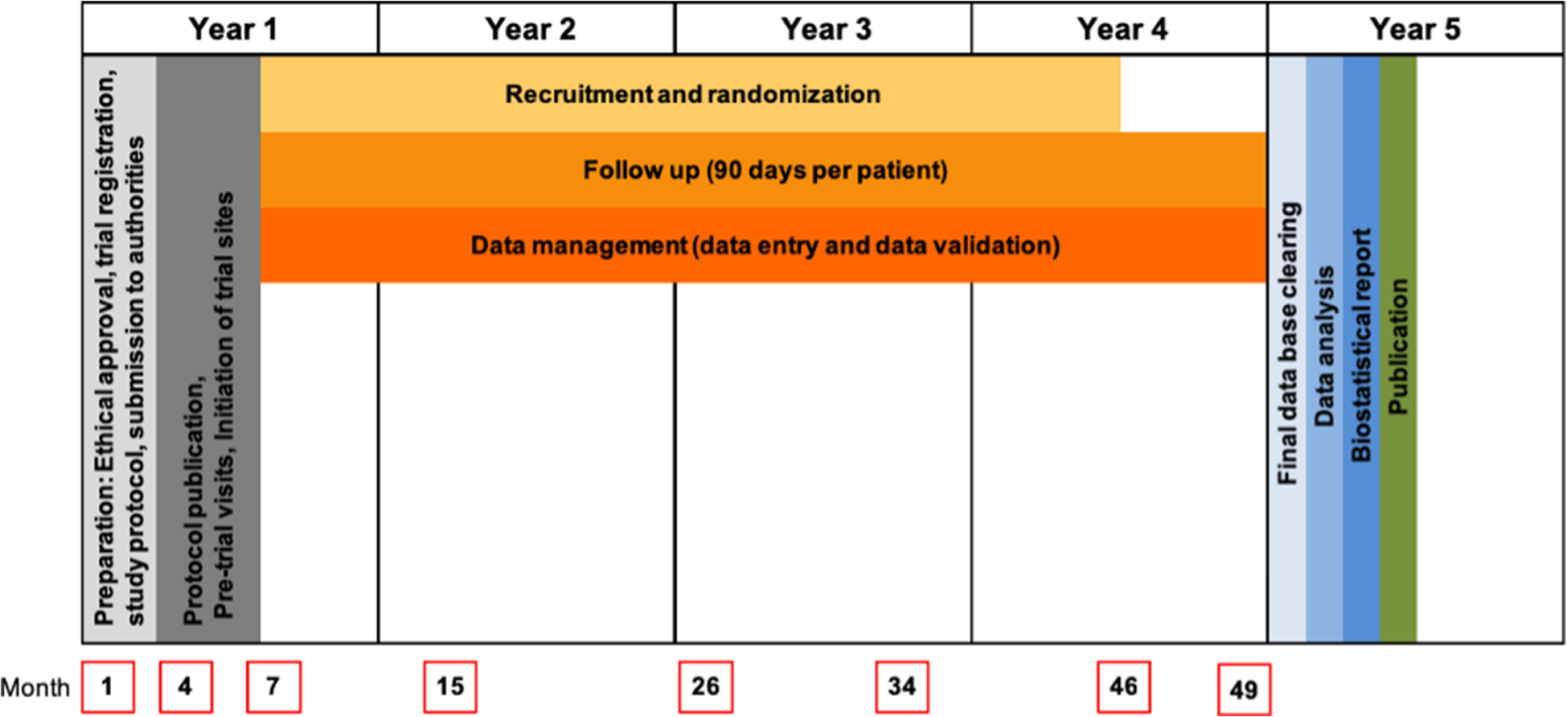
Trial timelines

Protocol Version: 3, Date: 25 February 2021. The duration of the clinical trial for each subject is expected to be 90 days. The overall duration of the clinical trial is expected to be approximately 54 months (details see Fig. 1) and the clinical phase 42 months. Recruitment of subjects will start in May 2021. The actual overall or recruitment duration may vary. Last subject out (LSO) is expected in October 2024.

## Abbreviations

AE: Adverse event
AP: Antibiotic prophylaxis
BDSG: Bundesdatenschutzgesetz (National Regulatory Requirements)
BfArM: Bundesinstitut für Arzneimittel und Medizinprodukte (Federal Institute for Drugs and Medical Devices, Germany)
CDC/KISS: Disease Control and Prevention criteria (CDC) and the “Krankenhaus-Infektions-Surveillance-System” (KISS)
CRP: C-reactive protein
CTFG: Clinical Trial Facilitation Group
DFG: Deutsche Forschungsgemeinschaft
DMC: Data monitoring committee
DRG: (German) Diagnosis-related groups system
eCRF: Electronic case report form
EC: Ethics Committee
ECDC: European Center for Disease Prevention and Control
EUDRACT: European Union Drug Regulating Authorities Clinical Trials
FCS: Fully conditional specification
GCP: Good Clinical Practice
GDPR: General Data Protection Regulation (EU Datenschutz-Grundverordnung (DSGVO))
HCG: Human Chorionic Gonadotropin (pregnancy hormone)
HRQoL: Health-related quality of life
ICH: International Council on Harmonisation of Technical Requirements for Registration of Pharmaceuticals for Human Use
ID: Identification
IMBI: Institute of Medical Biometry and Informatics
IMP: Investigational Medicinal Product
INN: International Nonproprietary Name
iv: Intravenous
KKS: Coordination Centre for Clinical Trials (Koordinierungszentrum für Klinische Studien)
LKP: Coordinating Investigator according to AMG (Leiter der Klinischen Prüfung)
LOS: Length of hospital stay
LSI: Last subject in
LSO: Last subject out
MIC: Minimal inhibitory concentration
OHIP-G 14: Oral Health Impact Profile (HRQoL questionnaire)
OS: Orthognathic surgery
POD: Postoperative day
RCT: Randomised controlled trial
SAE: Serious adverse event
SAP: Statistical analysis plan
SAS: SAS-software (SAS: Statistical Analysis Software)
SC: Steering Committee
SF-36: Short Form – 36 (HRQoL questionnaire)
SmPC: Summary of product characteristics
SOP: Standard operating procedure
SSI: Surgical site infection (defined by the CDC/KISS)
V: Visit
WBC: White blood cell
